# Antiviral Prophylaxis and Isolation for the Control of Pandemic Influenza

**DOI:** 10.3390/ijerph110807690

**Published:** 2014-07-31

**Authors:** Qingxia Zhang, Dingcheng Wang

**Affiliations:** School of Mathematical Sciences, University of Electronic Science and Technology of China, No. 2006, Xiyuan Avenue, West Hi-Tech Zone, Chengdu 611731, China; E-Mail: zqx121981@126.com

**Keywords:** mathematical modeling, pandemic influenza, antiviral prophylaxis, isolation

## Abstract

Before effective vaccines become available, antiviral drugs are considered as the major control strategies for a pandemic influenza. However, perhaps such control strategies can be severely hindered by the low-efficacy of antiviral drugs. For this reason, using antiviral drugs and an isolation strategy is included in our study. A compartmental model that allows for imported exposed individuals and asymptomatic cases is used to evaluate the effectiveness of control strategies via antiviral prophylaxis and isolation. Simulations show that isolation strategy plays a prominent role in containing transmission when antiviral drugs are not effective enough. Moreover, relatively few infected individuals need to be isolated per day. Because the accurate calculations of the needed numbers of antiviral drugs and the isolated infected are not easily available, we give two simple expressions approximating these numbers. We also derive an estimation for the total cost of these intervention strategies. These estimations obtained by a simple method provide a useful reference for the management department about the epidemic preparedness plans.

## 1. Introduction

The influenza virus, such as avian influenza H5N1 virus, is a great threat to public health [1,2,3]. Because we do not know what new influenza strain will appear in the future, it is possibly more dangerous and more difficult to contain its quick spread than any known influenza strain [4]. Vaccines are recognized as an effective means to prevent the spread of pandemic influenza; however, it takes about 180 days to develop an effective vaccine when a new strain of pandemic influenza arises [3,5,6]. Before effective vaccines become available, antiviral drugs, such as Tamiflu, and measures that reduce exposure to infected cases are used to mitigate the transmission of influenza virus [3,7]. To be specific, these emergency measures consist of avoiding close contact as much as possible, isolating infectious individuals in the hospital or at home, wearing personal protective equipment, such as masks and gloves, and the use of antiviral drugs for prevention and treatment [4,7]. 

Some previous work discussed the use of antiviral drugs for the control of influenza. Longini *et al*. [8] explored the effectiveness of targeted antiviral prophylaxis, in combination with other control measures. Black *et al*. [9] studied household-based interventions with the use of the antiviral drugs for the control of pandemic influenza. Antiviral drugs have been proven to be effective against currently circulating strains of pandemic influenza, so many countries set up a stockpile of antiviral drugs for preparedness plans [10,11,12,13,14]. However, the efficacy of antiviral drug against a new pandemic influenza strain is not yet known, and it is difficult to confirm whether current antiviral drug is effective or to what extent it is effective, against a novel influenza virus strain [14]. If the reproductive number cannot fall below one by using antiviral drugs, there will be a widespread outbreak of the disease. Therefore, it is very necessary to implement other control measures in conjunction with the use of antiviral drugs. 

Several previous studies have shown that the isolation strategy is an effective control measure. Sunmi Lee *et al*. [15] used optimal control theory to demonstrate that the isolation strategy has a prominent role when antiviral resources are limited. Yan *et al*. [16] showed that using isolation and quarantining strategies as much as possible at the start of the outbreak are very critical. 

Based on these considerations above, in this manuscript, we have incorporated the use of antiviral drugs and the isolation strategy in our model. We use a simple compartmental model to evaluate the effect of using antiviral drugs for prophylaxis and the isolation strategy to reduce transmission. It is impractical that antiviral drugs would be used only for prophylaxis and not for the treatment of cases, so we take into account antiviral prophylaxis supposing that antiviral drugs are used for treating index cases first, as Merler *et al*. [17]. We give two simple expressions, which approximate the needed numbers of antiviral drugs and the isolated infected, respectively, providing a practical reference for the sizes of antiviral stockpiles and healthcare resources (such as beds and personnel) for the possible epidemic in advance. Specifically, we consider the cost of these intervention strategies before the time when an effective vaccine against the new strain becomes available. We also give a concise expression, which approximates the total intervention cost. 

## 2. Methods

### 2.1. The Basic Model

**Figure 1:**
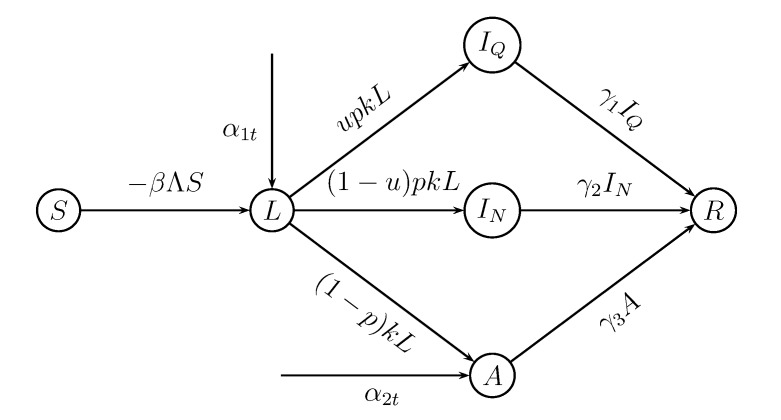
The summary of an influenza transmission model, where Λ= *f_m_*(*εI_Q_* + *I_N_*) + *σA*, *β* is the transmission rate. Exposed individuals leave the compartment at the rate *k*. A proportion 1 − *p* of infected individuals becomes asymptomatic cases and recovers in 1/*γ*_3_ days, and the rest of infected individuals develop symptoms. A fraction *u* of symptomatic infected individuals is isolated and recovers in 1/*γ*_1_ days; the remainder of symptomatic cases recover in 1/*γ*_2_ days. The rates of importing newly exposed persons and asymptomatic cases from other regions are *α*_1_*_t_* and *α*_2_*_t_*, respectively. The transmission rate can be reduced from *β* to *βf_m_* because of the use of antiviral drugs.

In this section, we introduce the influenza transmission model studied in this paper. This model is based upon a SLIAR model, which is an extended version of the standard SEIR model and is developed by Arino *et al*. [18], but includes the importing cases from other regions and interventions with isolation and the use of antiviral drugs. Figure 1 summarizes the model schematically. We consider a homogeneous population whose individuals mix uniformly and suppose that the importation approximately equals the exportation, neglecting births and natural deaths, so that the population remains almost unchanged. For simplicity, the mortality associated with the disease has not been included in our model explicitly, and the qualitative behavior of the model is not changed by this simplification [3]. 

We write *S* for the percentage of the people who are susceptible, *L* for the fraction of exposed individuals (*i.e.*, latent individuals—infected, but not infectious), *I_Q_* for the proportion who is symptomatic cases and has been isolated, *I_N_* for the proportion who is symptomatic cases, but not isolated, *A* for the fraction of the population who are asymptomatic, but infectious and *R* for the proportion who has recovered. The transmission and recovery are modeled by the following equations:

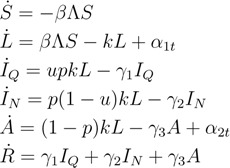
(1)
with Λ = *f_m_*(*εI_Q_* + *I_N_*) + *σA* (where *f_m_*, *ε* and *σ* are interpreted below) and initial conditions:

*S*(0) = *S*_0_, *L*(0) = *L*_0_, *I_Q_*(0) = *I_Q_*_0_, *I_N_*(0) = *I_N_*_0_, *A*(0) = *A*_0_, *R*(0) = *R*_0_

Susceptible individuals proceed to the latent compartment at the rate *β*(*f_m_*(*εI_Q_* + *I_N_*) + *σA*)*S*, where *β* is the transmission rate and $\dot{S}$ denotes *dS/dt*. We assume that infectivity and symptoms of influenza start at the same time [19], and each infected person who has obvious symptoms presents to the health service when his or her symptoms appear. As Becker and Wang [14], we suppose that each newly confirmed case causes the distributing of m doses of antiviral drugs to the case and persons who have close contact with that case. These drugs are used for treating the confirmed index case and providing prophylaxis to the associates of this index case [8,17]. For simplicity, in the following, the words antiviral prophylaxis mean that antiviral drugs are used for treating the confirmed index case and providing prophylaxis to the contacts of this index case. Since distributing the first few doses of antiviral drugs to the closest associates of the index case is probably more efficient in reducing transmission, as Becker and Wang [14], we adopt the following form of *f_m_*, which means that the transmission rate can be reduced from *β* to *βf_m_* by dispensing *m* antiviral doses per case, where:

*f_m_* = *a* + (1 − *a*)exp(−*bm*) (2)

for various values of *a* and *b* satisfying 0 ≤ *a* ≤ 1 and *b* > 0. Obviously, *f_m_* decreases from 1 to *a* with *m* increasing. 

Exposed members leave the compartment at the rate *k*, and a proportion *p* of exposed members develops symptoms, whereas the remainder proceed to an asymptomatic infectious compartment. A proportion *u* of symptomatic cases have been isolated and go to the recovered class at the rate *γ*_1_, while the rest of symptomatic cases recovered at the rate *γ*_2_. The infectivity of isolated cases is reduced by a factor of *ε*, with 0 ≤ *ε* ≤ 1. The case *ε* = 0 corresponds to the most perfect scenario when isolated cases have almost no contact at all with the susceptible individuals and the transmission rate is reduced to zero. The case *ε*> 0 represents the situation when contacts between isolated and susceptible individuals are not avoided. Gumel *et al*. [20] interpreted the modification parameter *ε* as the level of hygiene precautions during isolation. In fact, parameter f tunes the effectiveness of the isolation strategy, and it can somehow mimic the effect of a delay in the implementation of the interventions. The infectivity of asymptomatic individuals is reduced by a factor of *σ*, with 0 ≤ *σ* ≤ 1. In addition, asymptomatic individuals progress to the recovered compartment at the rate *γ*_3_. For simplicity, we assume that the transmission rate *β* and the recovery rates *γ*_1_, *γ*_2_, *γ*_3_ are not affected by seasonal variation. 

In addition, we suppose that symptomatic cases will not travel and that latent individuals are potentially traveling, because they have no symptoms [21]. We also assume that asymptomatic cases are potentially traveling. The model (1) includes an inflow of exposed individuals into the community at a rate *α*_1_*_t_* and an inflow of asymptomatic cases into the community at a rate *α*_2_*_t_*. Although the rate of importing exposed individuals and the rate of importing asymptomatic cases are time-dependent in practice, in this manuscript, we will concentrate on constant importation rates, *i.e.*, *α*_1_*_t_* = *α*_1_ and *α*_2__t_ = *α*_2_. 

### 2.2. Reproductive Numbers

In epidemiological models, the basic reproduction number is one of the most important quantities, denoted by *R*_0_ [22,23]. It is the average number of infections produced by an infectious individual in a wholly susceptible population when no public health interventions are implemented [23,24]. In the presence of control measures, it is called the control reproduction number [23]. In our model, the control measures include antiviral prophylaxis and isolation, and we denote the corresponding control reproduction number by *R_c_*. Applying the approach developed by van den Driessche and Watmough [25] to our model equations shows that the next-generation matrix *K* is:

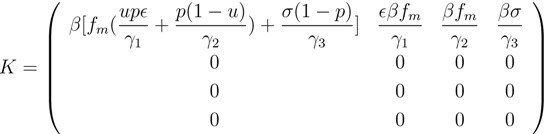
(3)
Since the rank of this matrix is 1, its spectral radius and trace are equal, which gives:

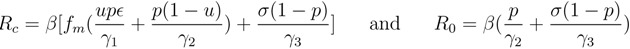
(4)


### 2.3. Intervention Cost for an Outbreak

Interventions can contain the spread of an outbreak, but they are costly. One of our aims is to derive an approximate expression for the intervention cost for an epidemic outbreak. Then, firstly, we need to model the cost of interventions. 

In this paper, we concentrate on two major intervention measures: antiviral prophylaxis and isolation. Following Ludkovski and Niemi [26], the total intervention cost is equal to a fixed cost plus a variable cost. A fixed cost is paid once when the intervention begins, and a variable cost is paid each time period and may depend on the latest number of actual infected individuals and isolated infected individuals. For simplicity, we assume the variable cost of antiviral prophylaxis is in proportion to the cumulative number of individuals who have received antiviral drugs up to time *t_v_*, *i.e.*,

(5) $$c_{1}\int_{0}^{t_{v}}mpkL(t)dt$$

where *t_v_* is the time when an effective vaccine against the novel strain of influenza can be supplied, the coefficient *c*_1_ is the cost per dose of antiviral drug and the integral expression:

(6) $$\int_{0}^{t_{v}}pkL(t)dt$$

represents the cumulative percentage of individuals who have received antiviral drugs up to time *t_v_*. The fixed cost of antiviral prophylaxis is denoted by *c*_3_(*t_v_*), which is a one-time upfront cost. Similarly, the variable cost of the isolation strategy is in proportion to the cumulative number of the infected who have been isolated up to time *t_v_*_, _*i.e.*,

(7) $$c_{2}\int_{0}^{t_{v}}upkL(t)dt$$

where the coefficient *c*_2_ is the cost of one infected individual who has been isolated during his or her infection period and the integral expression:

(8) $$\int_{0}^{t_{v}}upkL(t)dt$$

represents the cumulative percentage of the infected individuals who have been isolated up to time *t_v_*. The fixed cost of the isolation strategy is denoted by *c*_4_(*t_v_*), which is a one-time upfront cost. 

Then, the total intervention cost for an outbreak on [0, *t_v_*] is:

(9) $$c_{1}\int_{0}^{t_{v}}mpkL(t)dt+c_{2}\int_{0}^{t_{v}}upkL(t)dt+c_{3}(t_{v})+c_{\text{4}}(t_{v})$$

For simplicity, we take *c*_3_(*t_v_*) and *c*_4_(*t_v_*) to be constants and denote *c*_3_(*t_v_*) = *c*_3_, *c*_4_(*t_v_*) = *c*_4_. 

### 2.4. Approximating the Needed Number of Antiviral Doses Provided to Community Members, the Number of Isolated Infected Individuals and the Intervention Cost

Obviously, by numerical calculations, we can obtain the needed number of antiviral doses provided to community members, the number of isolated infected individuals and the intervention cost from Equations (1), (6), (8) and (9). It cannot be denied that the computational process is somewhat complex, and it is not intuitive and convenient for the management department. It is more instructive and convenient to work with concise expressions that approximate these numbers. This is because we can know clearly what factors influence these numbers and how these factors influence them, through these simple expressions. Next, we consider the approximate values of these numbers under the assumption that the epidemic can be contained. 

When *R_c_* is always smaller than 1, the depletion of susceptible individuals can almost be ignored, in other words, *S* ≈ 1. Inspired by the idea from [14], we may rewrite:

*L̇* ≈ *β*[*f_m_*(ε*I_Q_* + *I_N_*)+ *σA*] − *kL* + *α*_1_ (10)

By solving the simultaneous equations formed by the Equation (10) and the third to fifth equations in the model (1) with letting the right side of each equation be zero, we obtain the equilibrium value of the percentage of exposed individuals. The percentage of exposed individuals is close to its equilibrium value of (*α*_1_ + *βσα*_2_/*γ*_3_)/(*k*(1 − *R_c_*)). Replacing *L*(*t*) with its equilibrium value (*α*_1_ + *βσα*_2_/*γ*_3_)/(*k*(1 − *R_c_*)) in Equation (6), we can obtain that the approximate cumulative percentage of individuals who have received antiviral drugs up to time *t_v_* is *pt_v_*(*α*_1_ +*βσα*_2_/*γ*_3_)/(1 − *R_c_*). Therefore, the number of antiviral doses that need to be distributed to community members on [0, *t_v_*] is approximately:

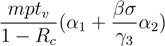
(11)
and the cost of antiviral prophylaxis is approximately:

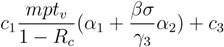
(12)
Similarly, by using Equation (8), we can obtain with the same methodology that the percentage of cumulative isolated infected individuals is approximately:

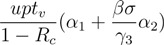
(13)
and the cost of the isolation intervention is approximately:

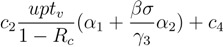
(14)
Then, the total intervention cost for an outbreak on [0, *t_v_*] is approximately:

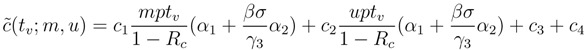
(15)


### 2.5. Parameters

Table 1 shows baseline values of all parameters of the model, where the values of *α*_1_, *α*_2_, *β*, *γ*_1_, *γ*_2_, *γ*_3_ and *k* are rates per day. We assume that a baseline initial value of *R*_0_ = 1.5, as in [14]. The latency period for influenza is supposed to be equal to the incubation period and is set at 1.5 days on average, namely *k* = 1/1.5 [17]. We also suppose that the possibility that an infected individual is developing symptoms of influenza is 0.5, namely *p* = 0.5 [17]. We further assume that symptomatic cases are twice infectious as those without symptoms, namely *σ* = 0.5 [8,17]. The recovery rate *γ_i _*= 1/1.5 (*i* = 1, 2, 3) means that the length of the infectious period is assumed to be 1.5 days [17,21]. The parameter *ε* = 1/6 assumes that the infectivity of isolated cases drops to 1/6 of the infectivity of symptomatic cases who are not isolated. The value *t_v_* = 180 days supposes that an effective vaccine will take 180 days to develop, manufacture and dispense, and *a* = 0.6 assumes that the effect of antiviral drugs is to reduce the risk of transmission per close contact by no more than 40%. In addition, we suppose that each person is initially susceptible, *i.e.*, *S*_0_ = 1 and *L*_0_ = *I_Q_*_0_ = *I_N_*_0_ = *A*_0_ = *R*_0_ = 0. The epidemic is seeded by the imported infectious individuals. Unfortunately, there are no good data on the cost coefficients *c*_1_, *c*_2_, *c*_3_ and *c*_4_; therefore, we give roughly the relative values of the cost coefficients. 

**Table 1:** Baseline values for model parameters.

***R*_0_**	***α*_1_**	***α*_2_**	***β***	***γ*_1_**	***γ*_2_**	***γ*_3_**	***p***
1.5	10^−6^	10^−6^	4/3	1/1.5	1/1.5	1/1.5	0.5
***k***	***σ***	***ε***	***t_v_***	***a***	***b***	***c*_1_**	***c*_2_**
1/1.5	0.5	1/6	180	0.6	0.2	1	50
***c*_3_**	***c*_4_**	***S*_0_**	***L*_0_**	***I_Q_*_0_**	***I_N_*_0_**	***A*_0_**	***R*_0_**
100	100	1	0	0	0	0	0

## 3. Results

### 3.1. Antiviral Drugs Prophylaxis and Isolation Strategy

Because effective vaccines will take about six months to produce once a novel influenza virus has been confirmed, the use of antiviral drugs is one of the most important intervention measures in the case of a pandemic [4,23]. We first consider that control strategy only via antiviral drugs for prophylaxis during an outbreak of pandemic influenza. 

**Figure 2:**
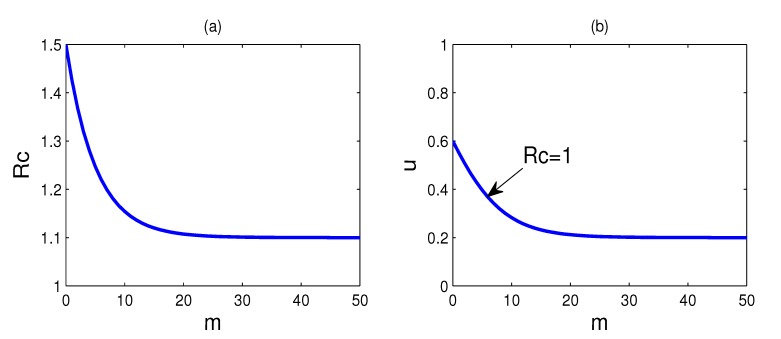
(**a**) The plot of the control reproduction number *R_c_* as a function of doses *m*, where *u* = 0 and other parameters assume the values of Table 1; (**b**) the plot for which *R_c_* is equal to one. Every parameter point (*m*, *u*) that corresponds to *R_c_* < 1 lies above this curve and *R_c_* > 1 for those points that lie below the *R_c_* = 1 curve.

Figure 2a shows the plot of the control reproduction number *R_c_* as a function of doses *m*, where *u* = 0 and other parameters suppose the values of Table 1. We see that the control reproduction number *R_c_* remains greater than one, even for substantial values of doses *m*. If *R_c_* is above one, the disease will widely spread in the population; hence, the successful containment of the epidemic is to reduce the reproduction number below one. It is impossible to contain the epidemic only by the use of the antiviral drugs if antiviral drugs have low-efficacy in reducing infectiousness against a new virus strain. Therefore, it is very necessary to implement other control measures in conjunction with the use of antiviral drugs. 

Isolation is one of the most effective methods to contain the spread of an outbreak [27]. For our purposes, we consider that the control strategy is taking the isolation strategy in conjunction with the use of antiviral drugs for prophylaxis. There are two possibilities when taking antiviral prophylaxis and the isolation strategy: *R*_0_ > *R_c_* > 1 and *R*_0_ > 1 > *R_c_*. The first corresponds to failure containment, and infected arrivals could lead to an outbreak. In this paper, we focus on the second scenario, *i.e.*, *R_c_* can be reduced to below one when taking antiviral prophylaxis and the isolation strategy widely and effectively. 

Figure 2b shows the values of *m* and *u* for which the control reproduction number *R_c_* is equal to one, namely *R_c_* = 1 for every parameter point (*m*, *u*) that lies on the curve. Meanwhile, every parameter point (*m*, *u*) that corresponds to *R_c_* < 1 lies above this curve and *R_c_* > 1 for those points that lie below this curve. By comparing the two curves in Figure 2, we can see that the reproduction number that is greater than one can be reduced to below one by the implementation of isolation of cases. Suppose the stockpile of antiviral drugs is sufficient; at least 20% of the infected individuals need to be isolated for reducing the reproduction number below one. On the other hand, at least 60% of the infected individuals need to be isolated if no antiviral drugs are provided. 

**Figure 3:**
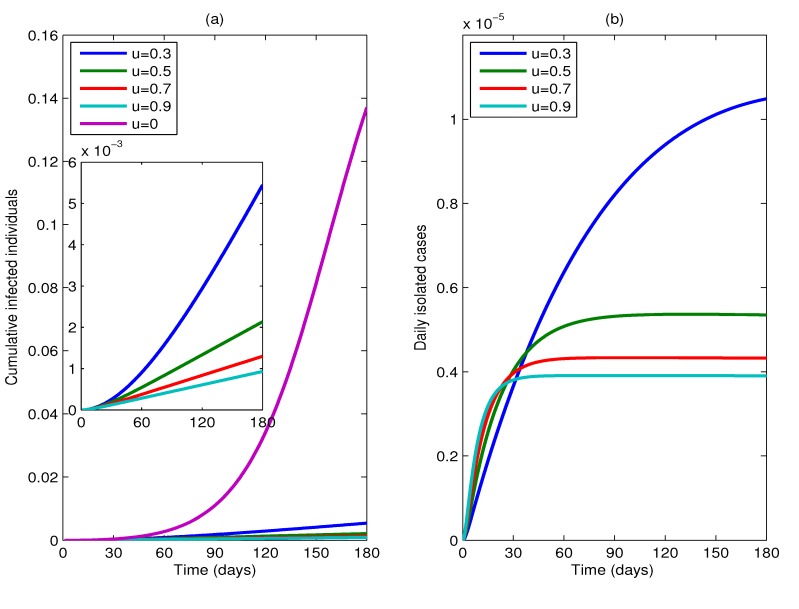
(**a**) Plots of the cumulative percentage of infected individuals up to time *t*; and (**b**) plots of the percentage of isolated cases *versus* time *t*, as predicted by the model (1) with parameters: *R*_0_ = 1.5, *a* = 0.6, *b* = 0.2, *m* = 15 and other parameters supposing the values of Table 1, for different values of *u*, where *u* = 0, 0.3, 0.5, 0.7, 0.9.

To provide a more intuitive result, Figure 3 shows plots of the cumulative percentage of infected individuals up to time t and the percentage of isolated infected individuals *versus* time t, respectively, as predicted by the model (1) with the parameter values of the model listed in Table 1, for different proportions of *u*, where *u* = 0, 0.3, 0.5, 0.7, 0.9 and *m* = 15. Comparing the curves in Figure 3a, we can see that taking antiviral prophylaxis combined with the isolation strategy can reduce substantially the cumulative number of infected individuals. At 180 days, the cumulative number of infected individuals when *u* = 0.3 drops to 1/25 of the cumulative number of infected individuals when no cases are isolated. By way of illustration, for a city with one million population members, allowing for one imported infective individual and one imported exposed individual per day, the cumulative number of infected individuals will be less than 2, 134 and the number of daily isolated cases will be less than six for a duration of six months if half of the symptomatic cases can be isolated. 

In addition, from Figure 3a, we can see that the cumulative percentage of infected individuals is always a monotonically decreasing function of *u*, *i.e.*, increasing the proportion of isolation can reduce the number of infected individuals; but the percentage of isolated infected individuals is only a monotonically decreasing function of *u*, after some fixed time point. This can be interpreted as follows. With the doses *m* fixed, *R_c_* decreases when the proportion *u* of isolation increases. Hence, in a short time horizon, the percentage of the isolated cases increases as *u* increases; but in a long time horizon, as *R_c_* decreases, the percentage of cumulative infected individuals decreases, and so does the percentage of the isolated cases. In conclusion, for containing the epidemic better, we need to keep *R_c_* under a level as low as possible, which implies that it is important to isolate infected individuals as much as possible in the early stage of the disease. 

From Figure 3b, we can see that as long as a few infected individuals need to be isolated every day and the reproductive number is reduced to below one, then the total number of the infected individuals can be reduced greatly (see Figure 3a). 

It is obvious from the above that implementing an isolation strategy has an obvious effect on the containment of the epidemic, especially if antiviral drugs have low-efficacy in reducing infectiousness. 

### 3.2. Comparisons between the Approximate Values and the Actual Values of the Percentage of Cumulative Symptomatic Cases, the Percentage of Cumulative Isolated Infected Individuals and the Intervention Cost

**Figure 4:**
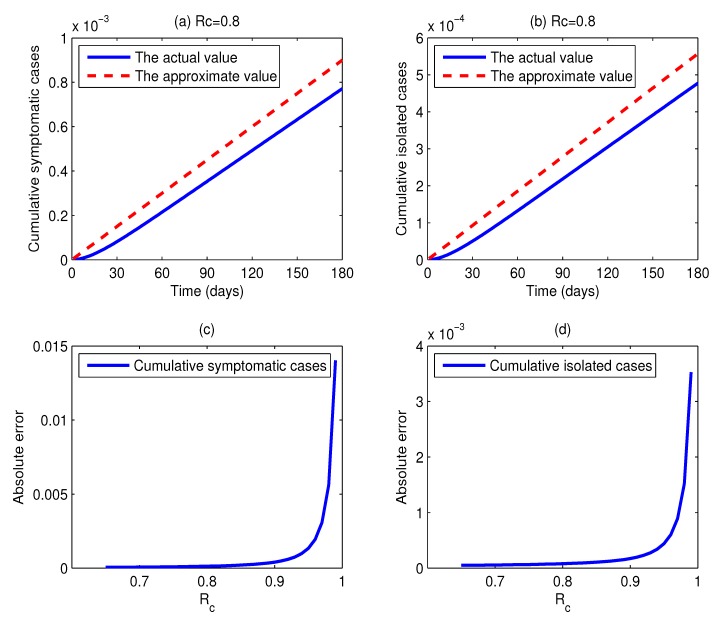
(**a**,**b**) Plots of the actual values and the approximate values of the cumulative percentages of infected individuals and isolated cases as functions of time *t*, respectively, where *R_c_* = 0.8, *m* = 15 and *u* =0.62; (**c**) plots of the absolute errors of the cumulative percentage of symptomatic cases as a function of *R_c_*; (**d**) plots of the absolute errors of the cumulative percentage of isolated cases as a function of *R_c_*.

Figure 4a,b shows the actual values and the approximate values of the cumulative percentages of symptomatic cases and isolated cases as functions of time *t* when *R_c_* = 0.8. Comparing the dashed lines with the solid lines in Figure 4, we can find that the dashed lines are located above the solid lines all of the time, but there is little difference between them, and they have the same change trends. This illustrates that the difference between the actual values and the approximate values is very little. More specifically, for a duration of 180 days, for every million individuals, the absolute error of the cumulative number of symptomatic individuals is 129, and the absolute error of the cumulative number of isolated cases is 80. Thus, in practice, we can take the approximate values as the estimations of the actual values, and the errors are quite small. On the other hand, the expressions of estimated values are simple and easy to be calculated. Based on the above merits, the approximate method is very useful for the management department to make control strategies. However, it should be noted that the absolute errors of the cumulative percentages of symptomatic individuals and isolated cases increase with the control reproduction number *R_c_* increasing. In other words, the smaller the control reproduction number *R_c_* is, the more accurate the approximation is. As we can see in Figure 4c, the curve begins relatively flat and after a while becomes very steep when *R_c_* exceeds 0.9. This implies that the approximation precision is relatively high if the reproduction number can be reduced below 0.9, and the approximation precision is significantly lowered when *R_c_* is over 0.9. The low accuracy may be caused by the approximation of *S* ≈ 1. The deviation between the percentage of susceptible individuals and one cannot be ignored when *R_c_* is close to one. 

**Figure 5:**
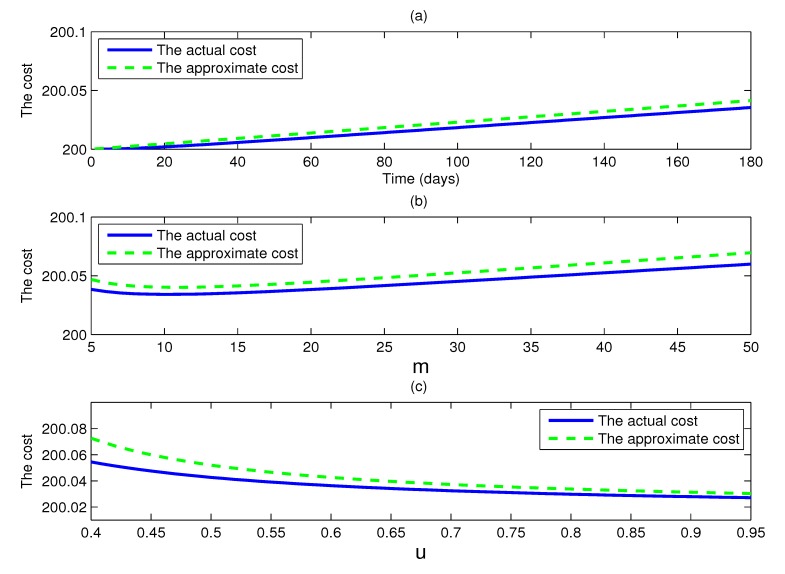
(**a**) Plots of the actual intervention cost and the approximate intervention cost as functions of time *t*, where *R_c_* =0.8, and the cost coefficients *c*_1_ = 1, *c*_2_ = 50, *c*_3_ = 100 and *c*_4_ = 100; (**b**) plots of the actual intervention cost and the approximate intervention cost as functions of doses *m*, where *R_c_* = 0.8 and the parameters as (a); (**c**) plots of the actual intervention cost and the approximate intervention cost as functions of *u*, where *m* = 15 and the parameters as (a).

Figure 5 shows the actual intervention cost and the approximate intervention cost as given by the model (1) with *c*_1_ = 1, *c*_2_ = 50, *c*_3_ = 100, *c*_4_ = 100 and other parameters supposing the values of Table 1. The three graphs all show that the difference between the actual intervention cost and the approximate intervention cost is very little. The reason is that the approximate values of the cumulative percentage of symptomatic cases and the cumulative percentage of isolated cases are very close to their actual values, and the cost is a linear combination of them, so the result is inevitable. 

At the same time, it is of interest to note that in Figure 5b, the actual intervention cost is not necessarily a monotonically increasing function of doses m, and there is a turning point in the actual intervention cost curve, where it is minimum. A similar situation takes place in the approximate intervention cost curve. For all parameter values, the actual intervention cost and the approximate intervention cost show a trend downward at first, when they reach their bottoms, and then begin to rise again, as *m* increases. This phenomenon tells us that in order to minimize the cost of intervention measures, a modest number of doses is enough, and too lager values of doses of antiviral drugs to reduce transmission only waste doses. 

These results have obvious implications for a public health service system. First, we give an estimation of the number of the cumulative isolated infected, so that the hospital can prepare the resources (such as beds and personnel) for the possible epidemic in advance. Second, the estimation of the required number of antiviral drugs provides a reference for the management department about the antiviral stockpile size for controlling the epidemic. This is very significant for the management department, because a small stockpile of antiviral drugs cannot satisfy the demand of the containment epidemic, whereas, a very large stockpile of antiviral drugs is a great waste of medical resources. Finally, the estimation of the cost of intervention will help the government with financial preparation prior to the possible pandemic influenza. 

### 3.3. Sensitivity Analysis

**Figure 6:**
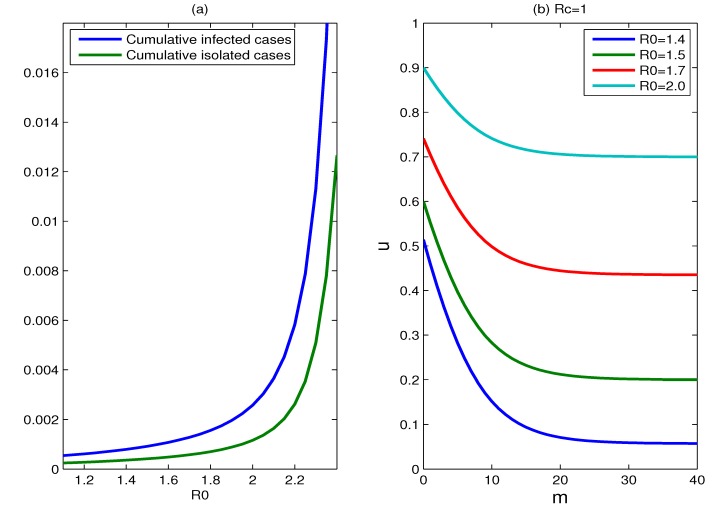
(**a**) Plots of the cumulative percentages of infected individuals and isolated cases during 180 days, as functions of *R*_0_, where *m* = 15, *u* = 0.9; (**b**) plots for which *R_c_* is equal to one for different values of *R*_0_, where *R*_0_ = 1.4, 1.5, 1.7, 2.0. Every parameter point (*m*, *u*) that corresponds to *R_c_* < 1 lies above this curve and *R_c_* > 1 for those points that lie below the *R_c_* = 1 curve.

**Figure 7:**
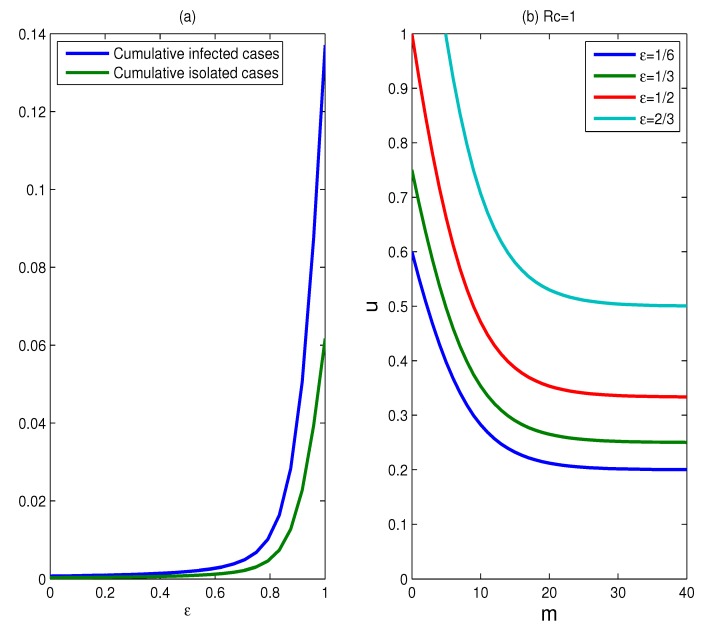
The effect of the infectivity reduction factor for isolated individuals. (**a**) Plots of the cumulative percentages of infected individuals and isolated cases as functions of *ε*, for a duration of six months, where *m* = 15, *u* = 0.9; (**b**) plots for which *R_c_* = 1, for various values of *ε*, where *ε* = 1/6, 1/3, 1/2, 2/3.

**Figure 8:**
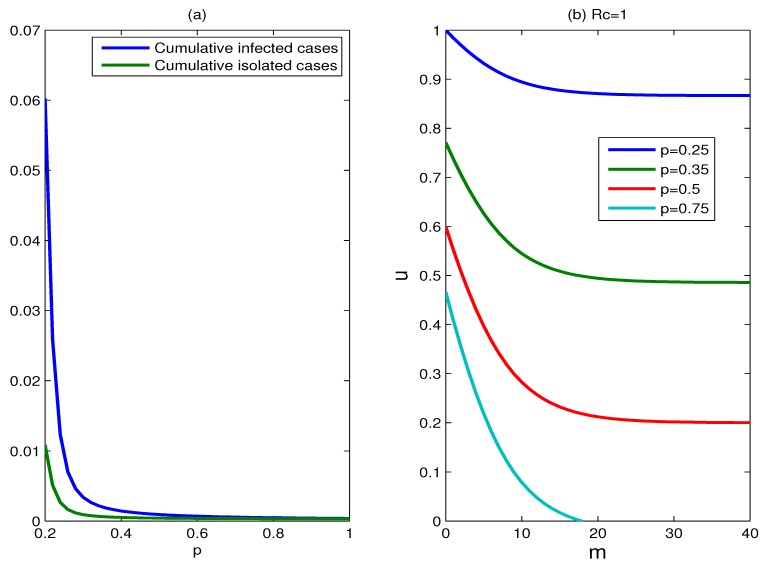
The effect of the proportion of symptomatic cases. (**a**) Plots of the cumulative percentages of infected individuals and isolated cases as functions of *p*, for a duration of six months, where *m* = 15, *u* = 0.9; (**b**) plots for which *R_c_* = 1, for various values of parameter *p*, where *p* = 0.25, 0.35, 0.5, 0.75.

**Figure 9:**
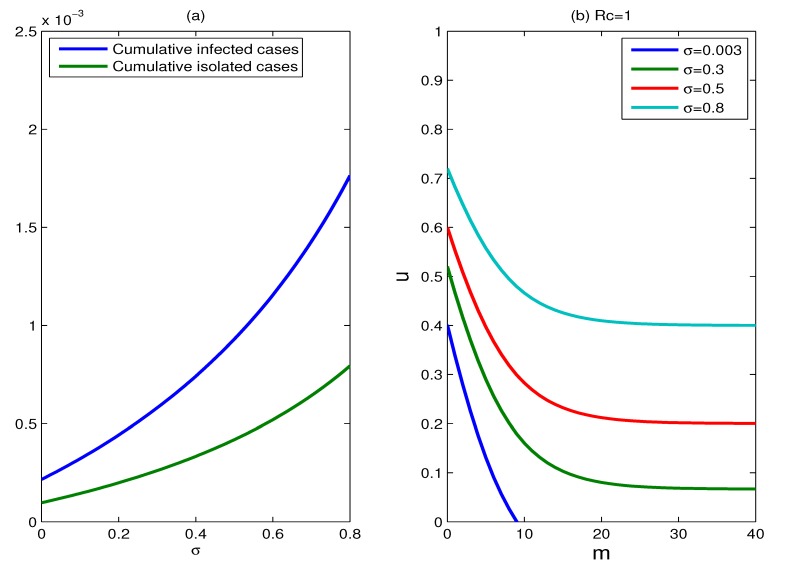
The effect of the relative infectivity of asymptomatic cases. (**a**) Plots of the cumulative percentages of infected individuals and isolated cases as functions of the parameter *σ*, for a duration of six months, where *u* = 0.9, *m* = 15; (**b**) plots for which *R_c_* = 1, for various values of parameter *σ*, where *σ* = 0.003, 0.3, 0.5, 0.8.

**Figure 10:**
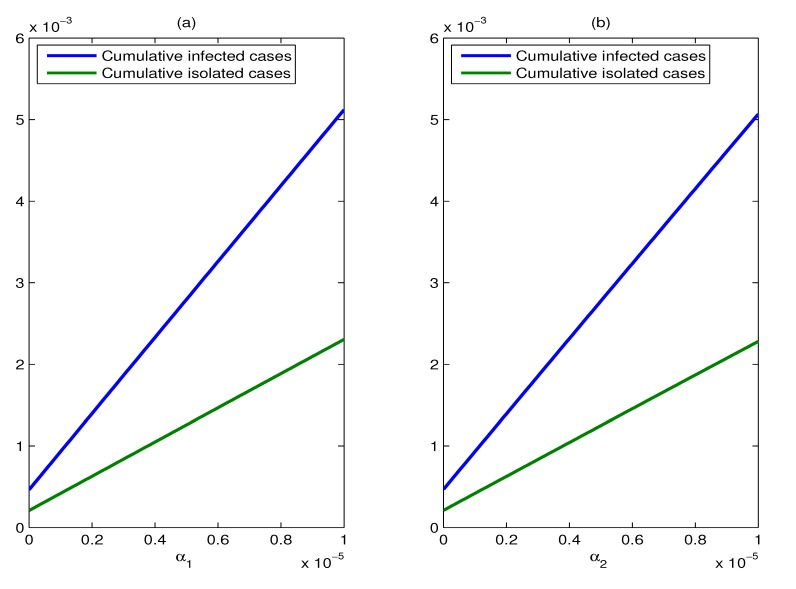
The effect of the imported rates of exposed individuals and asymptomatic cases. (**a**) Plots of the cumulative percentages of infected individuals and isolated cases as functions of α1, for a duration of six months, where *m* = 15, *u* = 0.9; (**b**) plots of the cumulative percentages of infected individuals and isolated cases as functions of α2, for a duration of six months, where *m* = 15, *u* = 0.9.

**Figure 11:**
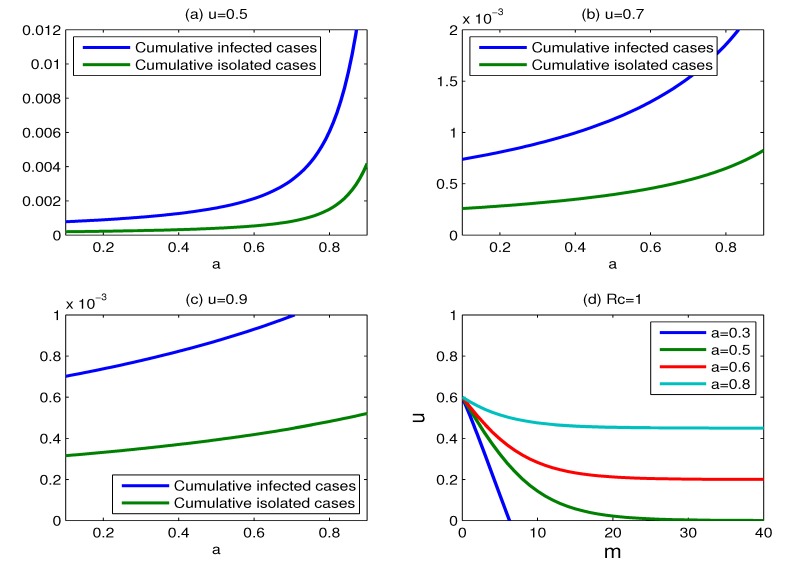
The effect of varying the efficacy of antiviral drugs. (**a**) Plots of the cumulative percentages of infected individuals and isolated cases as functions of the parameter *a*, where *m* = 15 and *u* = 0.5; (**b**) same as (a), but for *m* = 15 and *u* = 0.7; (**c**) same as (a), but for *m* = 15 and *u* =0.9; (**d**) plots for which *R_c_* = 1, for various values of parameter *a*, where *a* = 0.3, 0.5, 0.6, 0.8.

**Figure 12:**
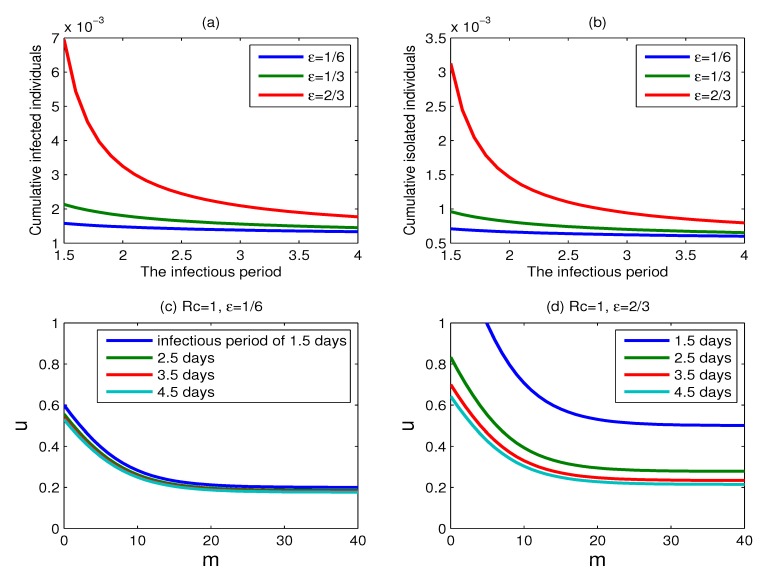
The effect of varying parameters determining infectious period. (**a**) Plots of the cumulative percentage of infected individuals as functions of the infectious period, for *ε*= 1/6, 1/3, 2/3, respectively; (**b**) same as (a), but for the cumulative percentage of isolated individuals; (**c**) plots for which *R_c_* = 1 for different infectious period 1/*γ_i_*, (*i* = 1, 2, 3) with *ε*= 1/6, where 1/*γ_i_* = 1.5, 2.5, 3.5, 4.5; (**d**) same as (c), but with *ε* = 2/3.

**Figure 13:**
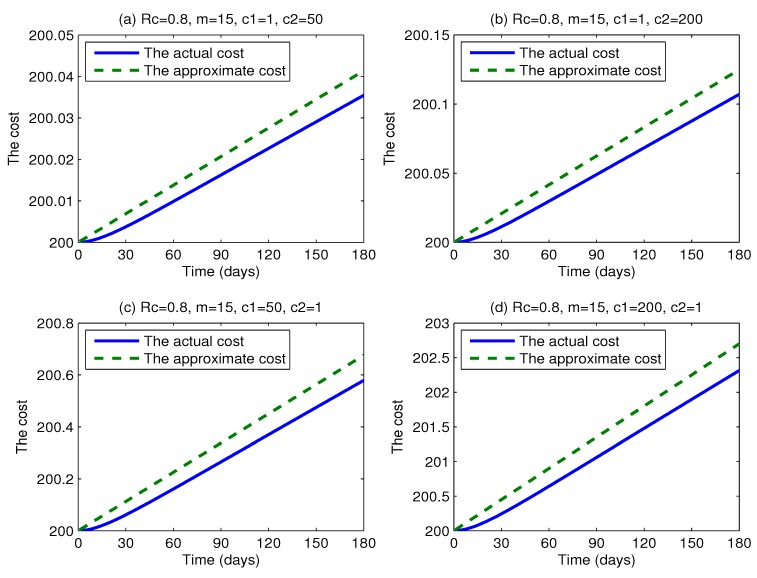
Plots of the actual intervention cost and the approximate intervention cost *versus* time t for various values of *c*_1_ and *c*_2_, where *c*_1_ = 1, 50, 200, *c*_2_ = 1, 50, 200, *R_c_* = 0.8, *c*_3_ = 100 and *c*_4_ = 100.

The baseline values of the parameters in Table 1 may not be suitable to a newly emerged strain of influenza virus. Therefore, we carry out a sensitivity analysis to investigate the effect of varying these parameters, such as the basic reproduction number (*R*_0_), the infectivity reduction factor for the isolated individuals (parameter *ε*), the proportion of symptomatic cases (parameter *p*), the relative infectivity of asymptomatic cases (parameter *σ*), the imported rate of exposed individuals (parameter *α*_1_), the imported rate of asymptomatic cases (parameter *α*_2_), the efficacy of antiviral drugs (parameters *a* and *b*), the infectious period (parameters 1/*γ*_1_, 1/*γ*_2_, 1/*γ*_3_) and the coefficients of the intervention cost (parameters *c*_1_, *c*_2_, *c*_3_ and *c*_4_). Figures 6, 7, 8, 9, 10, 11, 12, 13 show their impact on the results in Sections 3.1 and 3.2. When conducting the sensitivity analysis for a parameter, we assume the values of other parameters as Table 1, unless indicated otherwise. 

The effect of the basic reproduction number: Earlier in the manuscript, we supposed that the basic reproduction number *R*_0_ is 1.5. Figure 6a shows the effect of changing this assumption on the cumulative percentage of infected individuals. The cumulative percentage of infected individuals is quite sensitive to this change. For fixed parameters *m* and *u*, the cumulative percentage of infected individuals increases as *R*_0_ increases, and it increases rapidly when *R*_0_ surpasses some value. This is because the reproduction number cannot be reduced to below one for those values of *R*_0_ that exceed some value. The change of *R*_0_ gives the same impact on the cumulative percentage of isolated cases. Figure 6b shows plots for which *R_c_* is equal to one for different values of *R*_0_, where *R*_0_ = 1.4, 1.5, 1.7, 2.0. Every parameter point (*m*, *u*) that satisfies *R_c_* < 1 lies above the corresponding curve. The increase in the reproduction number *R*_0_ makes *R_c_* = 1 curve upward and shrinks significantly the set of parameter points for which *R_c_* < 1. This implies that it needs more effort to contain the transmission with larger *R*_0_. Moreover, the containment of the epidemic will not succeed if the reproduction number is too high, such as *R*_0_ > 2.5. 

The effect of the infectivity reduction factor for the isolated individual: The above results assume that the infectivity reduction factor (parameter *ε*) for the isolated individuals equals 1/6. The impact of altering this assumption is shown in Figure 7. The change of *ε* has a significant impact on the cumulative percentages of infected individuals and isolated cases (see Figure 7a). The low value of f results in a much less cumulative percentages of infected individuals and isolated cases, while larger f generates much more infected individuals and isolated cases. Especially, the slopes of the two curves suddenly grow larger when the value of *ε* exceeds 0.8, which means that the effectiveness of isolation strategies decreases significantly for *ε* > 0.8. This implies that the effectiveness of the isolation strategies is very sensitive to the parameter *ε*. Since the earlier the isolation begins, the better the effectiveness of the control strategies is, isolation strategies should begin as early as possible for better effectiveness of interventions. To obtain successful containment of the epidemic, the objective of the implementation of control measures is to bring *R_c_* below one. Figure 7b shows the curves for which *R_c_* is equal to one for different values of *ε* = 1/6, 1/3, 1/2, 2/3. As the parameter *ε* increases, the set of scenarios for which containment is achievable becomes smaller and smaller. Even containment will fail if the parameter *ε* is larger than some value. 

The effect of the proportion of symptomatic cases: Figure 8 shows the effect of varying the proportion of symptomatic cases (parameter *p*). The effectiveness of control measures is very sensitive to the proportion of symptomatic cases. For the fixed parameters *m* and *u*, the cumulative percentage of infected individuals decreases substantially with the proportion of symptomatic cases increasing. This is because more infected individuals are treated/isolated and more susceptible individuals receive antiviral prophylaxis. In Figure 8b, the set of scenarios for which containment is successful shrinks significantly as the proportion of symptomatic cases declines. If the proportion of symptomatic cases falls below a certain value (for example *p* < 0.2), the epidemic is hard to control. 

The effect of the relative infectivity of asymptomatic cases: The relative infectivity of asymptomatic cases (parameter *σ*) is studied in the range 0.003–0.8. The impact of varying the parameter *σ* on the cumulative percentages of infected individuals and isolated cases is shown in Figure 9a. Unsurprisingly, the effectiveness of control measures declines as the infectivity of asymptomatic cases increases. A low infectivity of asymptomatic cases (*σ* = 0.003) corresponds to a smaller cumulative number of infected individuals; conversely, a high infectivity (such as *σ* = 0.8) generates more infected cases. However on the whole, the effect caused by the change of *σ* is not very significant, since the two curves in Figure 9a always gently rise. Figure 9b shows the plots for which *R_c_* = 1, for different values of parameter *σ*, where *σ* = 0.003, 0.3, 0.5, 0.8. The set of scenarios for which containment is available becomes smaller as the values of parameter *σ* grows larger, and the reproduction number can be reduced below one for all the values of *σ* ranging from zero to one. 

The effect of the imported rates of exposed individuals and asymptomatic cases: We use three values for the imported rates of exposed individuals and asymptomatic cases ( *α_i_* = 10^−^^7^, 10^−^^6^, 10^−^^5^, *i* = 1, 2) to illustrate the effects of varying the assumed values of them. The change of the imported rate of exposed individuals makes no difference to the control reproduction number *R_c_*, according to the expression of *R_c_* (Formula (4)). However, this change has an impact on the cumulative percentages of infected individuals and isolated cases. It should be noted that for a fixed value of *α*_2_, the cumulative percentages of infected individuals and isolated cases increase linearly with the increase in the imported rate of exposed individuals (*α*_1_). From Figure 10b, we can see that the effect of varying the imported rate of asymptomatic cases (*α*_2_) is similar to that of varying the parameter *α*_1_. 

The effect of the efficacy of antiviral drugs: In Section 2.1, we assume that the effectiveness of dispensing *m* antiviral doses per case is to reduce the transmission rate from *β* to *βf_m_*, where *f_m_* = *a* + (1 − *a*)exp(−*bm*). The sensitivity of *b* has been discussed in the literature [14]. For large doses m, the effectiveness of antiviral drugs in reducing transmission largely depends on the parameter *a*. Therefore, we only consider the effect of varying the parameter *a*. The impact of the change of *a* on the cumulative percentages of infected individuals and isolated cases is shown in Figure 11. The cumulative percentages of infected individuals and isolated cases increase as the effectiveness of antiviral drugs declines for the fixed values of m and *u*. It is interesting to note that the influence of the change of *a* on the cumulative percentages of infected individuals and isolated cases becomes smaller with the proportion of isolation increasing. Figure 11d shows the curves for which *R_c_* = 1, for various values of parameter *a* = 0.3, 0.5, 0.6, 0.8. The set of scenarios for which containment is successful becomes large with the parameter *a* decreasing. In other words, containment is more likely to be successful when the value of *a* is small. Unfortunately, there is no good data to estimate the antiviral efficacy for a new pandemic strain before a control policy is instituted. In this case, regardless of whether antiviral drugs can effectively mitigate the transmission of a new pandemic strain, non-pharmaceutical measures, such as case isolation, combined with antiviral drugs, can be used to reduce the spread of influenza strain. 

The effect of the infectious period: The above results are based on the assumption that the infectious period is 1.5 days [17,21]. However, some models suppose that the infectious period is four days [3,14]. We carry out sensitivity analysis on the value of the infectious period. The impact of varying infectious periods on the effectiveness of control strategies is shown in Figure 12. The cumulative percentage of infected individuals is a little sensitive to the change of the infectious period with *ε* = 1/6, but it is very sensitive to the change of the infectious period when *ε* = 2/3 (see Figure 12a). The cumulative percentage of isolated individuals has the similar result as the cumulative percentage of infected individuals. Figure 12c,d shows the curves for which *R_c_* = 1 for different infectious periods 1/*γ_i_*, (*i* = 1, 2, 3) with *ε* = 1/6 and *ε*= 2/3, respectively, where 1/*γ_i_* = 1.5, 2.5, 3.5, 4.5 (*i* = 1, 2, 3). 

The set of scenarios for which containment is successful expands with the infectious period extending, and the expansion is more obvious for a larger value of parameter *ε*. 

The effect of the coefficients of the intervention cost: In order to make a sensitivity analysis on the coefficients of the intervention cost (parameters *c*_1_, *c*_2_) by comparing the actual cost with the approximate cost, we vary the coefficients *c*_1_ and *c*_2_, respectively. Figure 13 shows the actual intervention cost and the approximate intervention cost as functions of time t using four different coefficients (*c*_1_ = 1, *c*_2_ = 50; *c*_1_ = 1, *c*_2_ = 200; *c*_1_ = 50, *c*_2_ = 1; *c*_1_ = 200, *c*_2_ = 1, respectively). The two cost curves in every graph are quite close to each other. This illustrates that the changes of *c*_1_ and *c*_2_ could not have a great influence on the difference between the actual intervention cost and the approximate intervention cost. Obviously, the changes of *c*_3_ and *c*_4_ have little impact on the conclusion that there is little difference between the actual intervention cost and the approximate intervention cost. 

## 4. Discussion

One of our objectives was to see whether taking the isolation strategy should be included in an attempt to contain a newly emerged pandemic when antiviral drugs have low efficacy in reducing infectiousness against a new virus strain. The conclusion is a strong recommendation that implementing an isolation strategy is very effective if antiviral drugs are not effective enough. 

At the beginning of an outbreak, the efficacy of antiviral drug against a newly emerged pandemic influenza strain is not yet known, and it is difficult to confirm whether current antiviral drug is effective or to what extent it is effective. In this case, the implementation of the isolation strategy gives a chance to restrict the impact of pandemic influenza by lowering infection rates. Therefore, the isolation strategy is always effective for the uncertainty of the efficacy of the antiviral drugs against a newly emerged influenza strain. In addition, sensitivity analysis implies that the effectiveness of the isolation strategies is very sensitive to the parameter *ε*. Since the earlier the isolation begins, the better the effectiveness of the control strategies is, isolation strategies should begin as early as possible for better effectiveness of interventions. The large fraction of asymptomatic individuals is one of the key features of influenza; Hayward *et al*. [28] pointed out that no symptoms were present in three quarters of the infected individuals for the 2009 H1N1 pandemic. We therefore consider those infected individuals who have no symptoms. The conclusion is that containment is not successful if the proportion of asymptomatic cases exceeds a specific value, even though the intervention strategies are efficient enough. In other words, if taking antiviral prophylaxis and the isolation strategy cannot contain transmission at the beginning of an outbreak, the most likely reason for this failure is that a large number of infected individuals fail to display symptoms. In this case, the most effective measure for containing the epidemic may be to isolate the symptomatic cases who present to the health services and place the closest associates of the symptomatic cases into self-isolation, because they are possible asymptomatic cases. In short, the government should focus on antiviral prophylaxis and the isolation strategy in the early stage of an outbreak, and after a while, if containment is not achievable and the strain continues to spread, other intervention measures (such as self-isolation [27]) should be considered. This problem will be further explored in future work. 

Because the accurate calculations of the needed numbers of antiviral drugs and the isolated infected are not easily available, we give two simple expressions approximating these numbers. We also derive an estimation for the total cost of these intervention strategies. These estimations that are gotten by a simple method provide a useful reference for the management department about the epidemic preparedness plans. These conclusions depend largely on the condition *R_c_* < 1 (which means successful containment), and these results can be applied to a wide range of disease characteristics. This methodology still can be used to approximate other intervention costs, only if the intervention strategy can reduce the reproduction number to less than one. 

In addition, in order to confirm the effectiveness of antiviral drugs against the novel influenza virus strain, we must collect some data at the beginning of the local outbreak. According to the result in [14], the data on 100–200 household outbreaks are needed to examine an antiviral effect on transmission. 

On the basis of a simple compartmental model, some practical results have been obtained. These results are very meaningful and important for policy makers in making intervention 
